# Characterization of fungal communities on shared bicycles in Southwest China

**DOI:** 10.1186/s12866-021-02338-4

**Published:** 2021-10-18

**Authors:** Lu Peng, Bi Qin, Zhu Shen, Siyu Wang

**Affiliations:** 1 Department of Dermatology, Institute of Dermatology and Venereology, Sichuan Academy of Medical Sciences & Sichuan Provincial People’s Hospital, No.32, Western 2nd Section, 1st Ring Rd, Qingyang District, Chengdu, 610072 Sichuan China; 2 School of Medicine, University of Electronic Science and Technology of China, Chengdu, 610054 China; 3 Department of Dermatology, Acupuncture & Moxibustion Research Institute, Sichuan Academy of Traditional Chinese Medicine, Sichuan Second Hospital of Traditional Chinese Medicine, Chengdu, 610031 Sichuan China

**Keywords:** Shared-bicycle, Fungal communities, Potential pathogens

## Abstract

**Background:**

The widespread use of shared bicycles has increased the demand and sanitary requirements for shared bicycles. Previous studies have identified potentially pathogenic bacteria on the surfaces of shared bicycles, but fungal communities have not been investigated.

**Methods:**

We sampled shared-bicycle handles and saddles from five selected locations in a metropolis (Chengdu, China, *n* = 98) and used surrounding air deposition samples as controls (*n* = 12). Full-length ITS sequencing and multiple bioinformatic analyses were utilized to reveal fungal community structures and differences.

**Results:**

*Aspergillus* was dominant on both the handles and saddles of shared bicycles, and *Alternaria* and *Cladosporium* were the most abundant families in the air samples. Significant differences in fungal community structures were found among the three groups. The handle samples contained higher abundances of *Aureobasidium melanogenum* and *Filobasidium magnum* than the saddle and air samples. The saddle samples had a higher abundance of *Cladosporium tenuissimum* than the other two sample types (*P* < 0·05). A higher abundance of fungal animal pathogens on shared-bicycle surfaces than in air by FUNGuild (*P* < 0·05). Moreover, the co-occurrence network of fungi on handles was more stable than that on saddles.

**Conclusion:**

There were more potential pathogens, including *Aspergillus pseudoglaucus*, *Aureobasidium melanogenum*, *Kazachstania pintolopesii*, *Filobasidium magnum*, *Candida tropicalis*, and *Malassezia globose* were found on shared bicycles than in air, suggesting that hands should not contact mucous membrane after cycling, especially in susceptible individuals, and hygiene management of shared bicycles should be given more attention by relevant organizations worldwide.

**Supplementary Information:**

The online version contains supplementary material available at 10.1186/s12866-021-02338-4.

## Introduction

Shared bicycles, as a widely implemented public health project, have been made available in more than 1000 cities in the past decade. Shared bicycles has changed the travel mode of the urban population to some extent and have gradually become indispensable [1]. In recent years, especially during the coronavirus disease 2019 (COVID-19) pandemic, the demand for shared bicycles increased due to their convenience and environmental friendliness in cities with large populations. As a public transportation tool, shared bicycles are stored in outdoor public areas for long periods of time and repeatedly contacted by different people and their clothes, which harbor different microbes, including bacteria and fungi. The microbes on a shared bicycle will change and further spread during consecutive use. Potentially pathogenic bacteria [2] and antimicrobial-resistant *Enterobacteriaceae* [3] have been sequenced from samples from shared bicycles. However, to date, no study has yet focused on fungi on shared bicycles. Fungi are the most common microorganisms in the environment; types of fungi can vary widely and be potentially pathogenic in humans under certain circumstances. Thus, our study is the first to analyze of the structure and function of fungi on the shared bicycles.

## Method

### Sample collection

Swab samples of saddle and handle surfaces were collected from a number of shared bicycles in five locations in a metropolitan area (Chengdu, China) in July 2020. Samples from shared bicycles that had gone unused for a long time or were obviously damaged were excluded. Random samples from nearby air sampling sites were collected on the same day in each selected area. The samples were collected from the handles and saddles of the shared bicycles (approximately 10 cm [2]) with DNA-free swabs (Puritan, Me, USA), which were applied for 15 s. Each surface was sampled with a new cotton swabs and rotated three times in a nonoverlapping area; sampling was repeated three times. Air samples were collected by a sterile filter paper in a petri dish, which was placed at a high location in the selected area for 6 h. After sampling, each sample was stored in a unique labeled sterile centrifuge tube and transported to the laboratory on dry ice before being immediately stored in a refrigerator at − 80 ° Celsius for further analysis.

### DNA extraction and internal transcribed spacer (ITS) amplification sequencing

Genomic DNA from samples collected from the different sampling sites and surfaces was extracted by the CTAB/SDS method. The concentration and purity of the extracted DNA was measured with a 1% agarose gel and then diluted with 1 ng/μl sterile water.

The ITS region was amplified by specific primers with barcodes and Phusion® high-fidelity PCR master mix with GC buffer (New England Biolabs). The PCR products were purified with a QIAquick@ Gel Extraction Kit (QIAGEN) after mixing with the same volume of 1X loading buffer and detection by electrophoresis in a 2% agarose gel.

Following the manufacturer’s instructions, sequencing libraries were generated using the SMRTbellTM Template Prep Kit (PacBio). After assessment using a Qubit® 2.0 fluorometer (Thermo Scientific) and a FEMTO Pulse system, the library was sequenced on the PacBio Sequel platform.

We corrected raw sequence reads using circular consensus sequencing (CCS, SMRT Link version 7.0), with 3 passes and a minimum predicted accuracy of 99% [4]. After obtaining denoised FASTQ sequences of expected amplicon size (minLength 500 bp, maxLength 1000 bp) and removing chimeras by simple sequence repeat (SSR) detection, we used ITSx software (http://microbiology.se/software/itsx) to extract the full-length sequences of filtered reads as clean reads.

### Operational taxonomic unit (OTU) clustering and species annotation

We used Uparse software (Uparse version 7.0.1001, http://drive5.com/uparse/) to assign sequences with ≥97% similarity to the same OTU. Then, we selected the sequence with the highest frequency in each OTU as a representative sequence, which was further annotated using the BLAST method (http://qiime.org/scripts/assign_taxonomy.html) based on QIIME software (version 1.9.1) and the Unite database (https://unite.ut.ee/) [5]. After data analysis, we acquired the OTU abundance at the phylum, class, order, family, genus, and species levels for each sample. The phylogenetic relationship of all the representative OTUs was assembled by MUSCLE software (version 3.8.31, http://www.drive5.com/muscle/).

### Fungal community structure analysis

Before subsequent analysis, OTU abundance data were normalized to the fewest sequences. Alpha diversity indices, including the Chao 1, abundance-based coverage estimator (ACE), and Shannon indices, were calculated using QIIME (version 1.9.1) and R software (version 2.15.3). Differences in alpha diversity between groups were analyzed by the Wilcox test, and the significance threshold was set at 0·05.

Beta diversity, including weighted UniFrac and unweighted pair-group methods with arithmetic mean (UPGMA) clustering, was calculated by QIIME software (version 1.9.1). Principal coordinate analysis (PCoA) was performed to obtain principal coordinates and visualize complex, multidimensional data by R software (FactoMineR package, WGCNA package, stat packages and ggplot2 package, version 2.15.3).

Linear discriminant analysis effect size (LefSe) and MetaStat were used to identify the taxa that were differentially abundant among the shared-bicycle surfaces and the air. A linear discriminant analysis score cutoff of 4·0 and other parameters were set as defaults. For the MetaStat analysis, species with significant differences between groups were analyzed with R software (version 2.15.3), and a *p* value less than 0·05 was set as the significance threshold.

For functional analysis of fungal communities, we taxonomically parsed fungal OTUs into trophic modes and guilds using FUNGuild [6]. To ensure validity, we retained only the guilds with confidence ratings of “probable” and “highly probable.”

Co-occurrence networks between genera from the samples of bicycles were constructed and visualized in GraphViz software (version 2.38.0). Spearman’s rank correlations between selected genera were calculated using an R package. A valid co-occurrence was considered to have a strong correlation if Spearman’s correlation coefficient (ρ) was greater than 0·6, with a corrected significance level less than 0·01.

## Results

### Sampling of shared-bicycles and surrounding air

We collected samples (*n* = 130) from several shared bicycles and air in five selected locations (central, eastern, western, southern and northern areas) of a metropolis (Chengdu, China) for fungal community analysis. The central location in Chengdu was near general hospitals, and the eastern location was in a residential area; in contrast, the western location was near busy commercial streets. The southern and northern locations were close to a senior high school and metro station, respectively. A total of 110 qualified DNA samples were obtained, including samples from shared bicycles (*n* = 98) and the air around them (A group, *n* = 12). Shared-bicycle samples were collected from the handles (H group, *n* = 50) and saddles (S group, *n* = 48).

Eligible DNA extracted from these samples was subjected to ITS sequencing analysis. After stringent-quality sequencing and filtering, we obtained a total of 1,321,604 clean reads (82% of the total 1,608,556 raw reads) from the 110 samples. Then, 3724 unique OTUs were clustered, and 717 genera and 49 classes from 14 fungal phyla were identified by the UNITE database; 1·1% of the OTUs could not be matched to taxa in the database.

### Fungal community composition

**Fig. 1:**
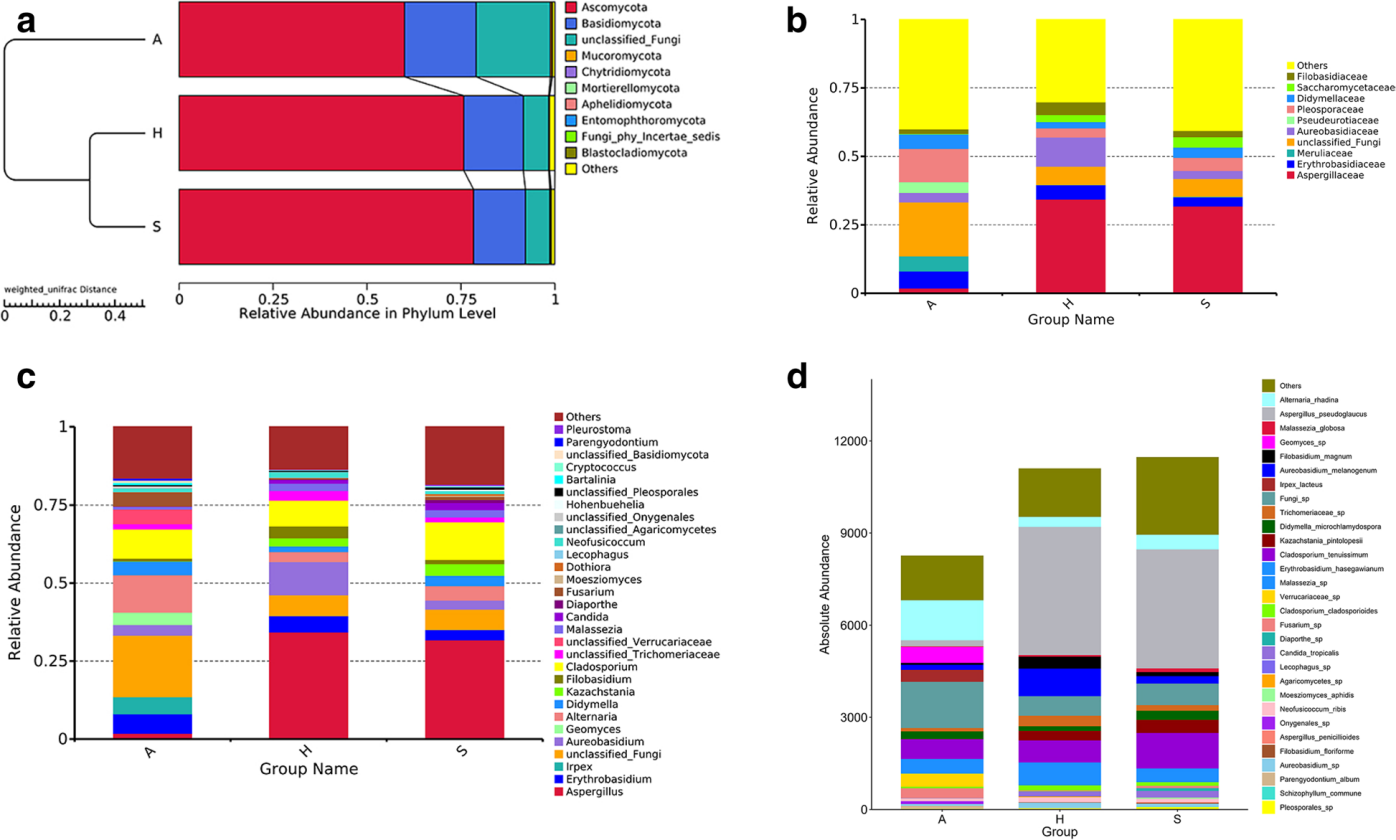
Fungal community composition on shared bicycles and in nearby air. The weighted UniFrac UPGMA distance matrix with the top ten most abundant fungi at the phylum level is on the right (**A**); the top 10 most abundant fungi at the family level (**B**), the top 30 most relative abundant fungi at the genus level (**C**), and the top 30 most absolute abundant fungi at the species level (**D**). (A: air, H: handle, S: saddle)

The fungal community constituents of the three groups were observed at the phylum level (Fig. 1A). The relative abundances of Ascomycota and Basidiomycota were markedly dominant, at 75·7% and 15·9% on handles, 78·4% and 13·8% on saddles, and 60·1% and 19·0% in air samples, respectively. An unclassified fungal phylum with a relative abundance of 20·0% was identified in the air group. The similarity between the saddles and handle samples was reconfirmed by the weighted UniFrac UPGMA distance matrix.

Aspergillaceae had the highest abundance among the top ten families in the shared-bicycle samples (handles and saddles) (Fig. 1B), followed by Aureobasidiaceae in handle samples and unclassified fungi in saddle samples. These results differed from those of the air samples, which were characterized by a relatively high abundance of unclassified fungi and Pleosporaceae, which accounted for approximately 19·7% and 12·2%, respectively.

The relative abundances of the top 30 fungal constituents at the genus level in the three groups also varied (Fig. 1C, see Additional Fig. 1). Excluding unclassified fungi*,* five genera, namely, *Aspergillus*, *Candida*, *Alternaria*, *Cladosporium* and *Erythrobasidium*, were enriched in the handle samples. *Aspergillus* and *Cladosporium* also dominated the mycobiota in the saddle samples. The fungal communities in air samples were more homogeneous; *Alternaria* and *Cladosporium* had the highest abundances, followed by *Irpex* and *Erythrobasidium*.

At the species level, the absolute abundance of *Aspergillus pseudoglaucus* was overrepresented in the handle and saddle samples. *Alternaria rhadina* was the most abundant species in air, followed by *Cladosporium tenuissimum* and *Erythrobasidium hasegawianum,* which were highly abundant in all three groups. In addition, *Aureobasidium melanogenum* and *Filobasidium magnum* were the main species on handles. *Cladosporium tenuissimum*, *Alternaria rhadina*, and *Kazachstania pintolopesii* were the main species on saddles. *Malassezia sp., Candida tropicalis, Malassezia globose* and *Trichomeriaceae sp.* were also included in the top 30 most abundant species (Fig. 1D).

### Fungal alpha diversity

**Fig. 2:**
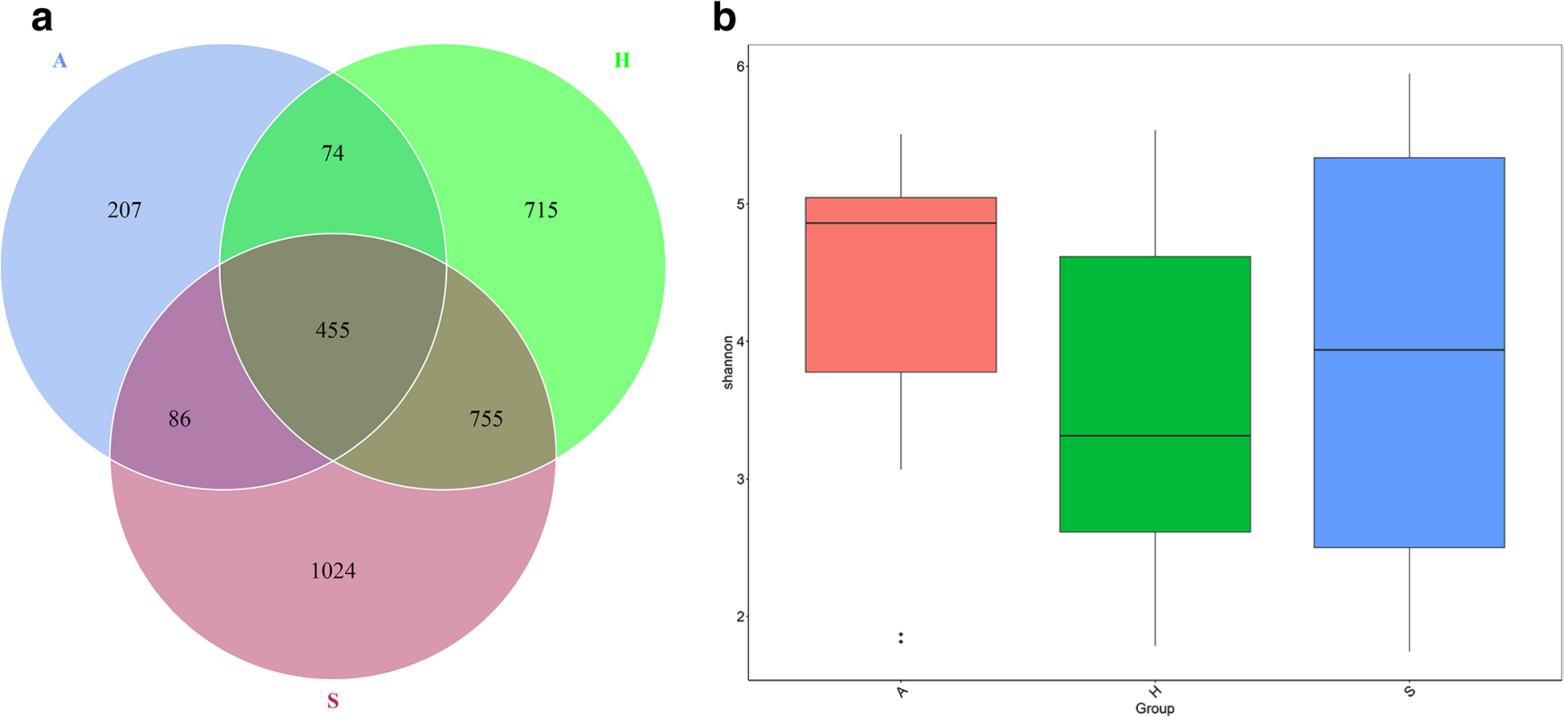
Fungal alpha diversity of shared bicycles and nearby air. Venn diagram based on OTUs detected on shared bicycles and in nearby air (**A**), Shannon index for shared bicycles and nearby air, positive correlation with diversity (**B**). (A: air, H: handle, S: saddle)

The highest community richness was found on saddles, and the lowest richness was found in air (Fig. 2A; see Additional Figs. 2 and 3). Species were more similar between handles and saddles, with three and five times the number of endemic OTUs than air, respectively. The proportions of OTUs shared by the air and bicycle surfaces were 19·9% (615/3109), 26·5% (529/1999) and 23·3% (541/2320) for the combined surfaces, handles, and saddles, respectively. Combined with richness and evenness, the Shannon index (Fig. 2B) indicated a more complex fungal community diversity in air than on shared bicycles. However, there were no significant differences in the species richness and Shannon diversity indices among the three groups.

### Fungal beta diversity

**Fig. 3:**
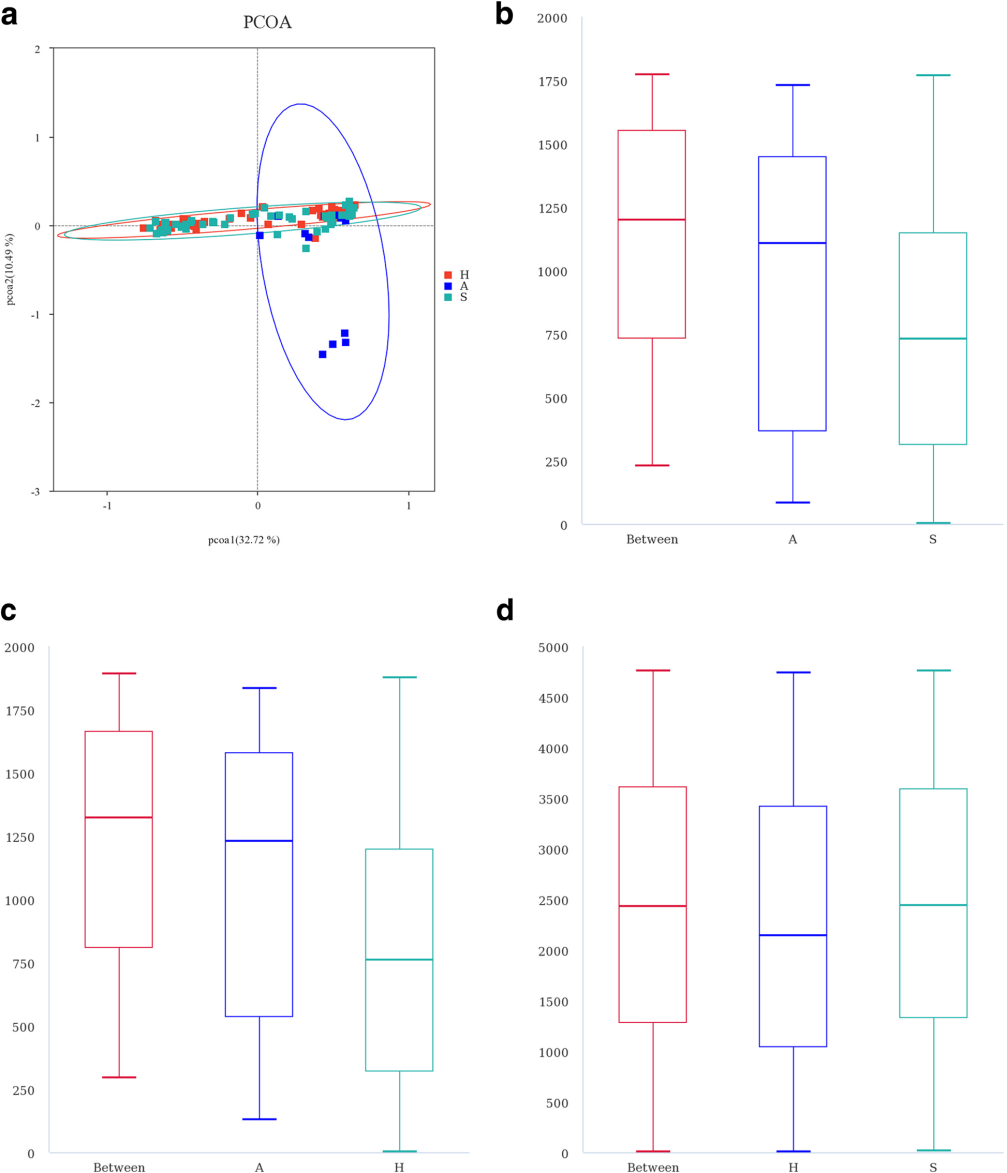
Fungal beta diversity of shared bicycle and nearby air samples. Weighted UniFrac distance-based PCoA. The percentage represents the contribution of the principal component to the sample difference. Each diamond in the diagram represents a sample, and H and S samples tend to cluster together and away from group **A**, indicating that the fungal community structure on Hs and Ss is highly similar and greatly different from that of A. ANOSIM intergroup difference analysis. The ordinate represents the rank of the distance between samples, “Between” in the abscissa represents the results between two groups, and the other two represent the results within each group (**B**-**D**). (A: air, H: handle, S: saddle)

In the PCoA based on weighted UniFrac distances (Fig. 3A), the OTUs from handle and saddle samples tended to cluster together and were more distant from those from air samples. This indicates that the fungal community structures on handles and saddles are highly similar to each other and different from that in air. In addition to confirming the above conclusion, ANOSIM analysis (Fig. 3B-D) demonstrated a significant differences in the fungal community structure between the bicycle surfaces (R > 0).

### Comparisons between groups

**Fig. 4:**
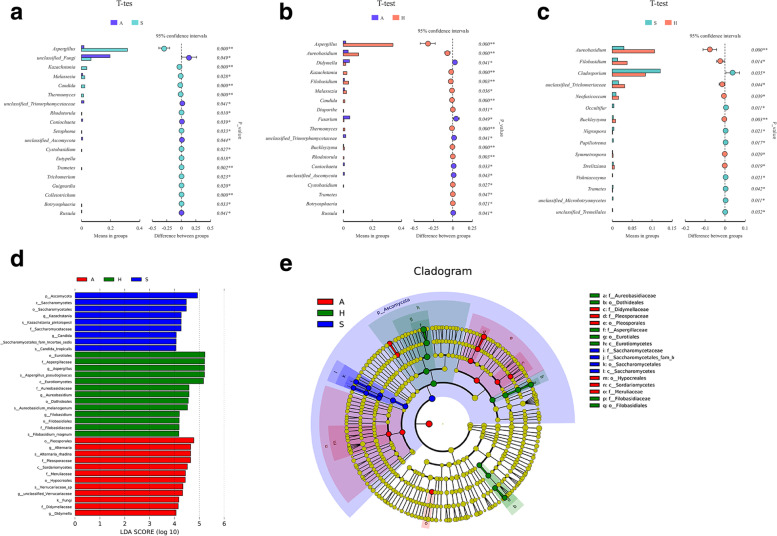
Fungal diversity comparison between shared bicycles and nearby air. T-test at the genus level (**A**-**C**). LEfSe of significantly different taxa between groups, linear discriminant analysis (LDA) value distribution, (LDA Score > 4) (5 D), evolutionary branch (5E). (A: air, H: handle, S: saddle)

After the above diversity analysis, we focused on differential abundances in fungal communities among the three groups. The t-test and MetaStat analysis (Fig. 4A-C; see Additional Fig. 4) between groups indicated significant differences in community structure between not only the shared bicycles and air samples but also the handles and saddles at the genus and species levels, even though the diversity on the handles and saddles had greater similarity.

Compared with the air samples, the shared bicycle (handle and saddle) samples showed significant enrichment of the genera *Aspergillus*, *Kazachstania*, *Candida* and *Malassezia* (Fig. 4A-B, *P* < 0·05). It is worth noting that certain species, such as *Aspergillus pseudoglaucus*, *Kazachstania pintolopesii*, *Candida tropicalis*, and *Malassezia globose,* are likely responsible for the differences observed in abundances at the genus level (see Additional Fig. 4).

The abundances of *Aureobasidium* and *Filobasidium* were more enriched in the handle group than in both the saddle and air groups, while *Cladosporium* was more enriched on saddles (Fig. 4A-C, see Additional Fig. 4, P < 0·05). Similarly, the differences in these genera are likely primarily attributed to the high relative abundance of *Aureobasidium melanogenum* and *Filobasidium magnum* on handles and *Cladosporium tenuissimum* on saddles.

The evolutionary branches of the LEfSe analysis indicated that the fungal markers with significant differences among the three groups originated from 8 genera of 3 classes (*Saccharomycetales*, *Eurotiomycetes,* and *Sordariomycetes*) (Fig. 4D). The 8 genera were *Kazachstania* and *Candida* in the saddle samples; *Aspergillus*, *Aureobasidium* and *Filobasidium* in the handle samples; and *Alternaria, Didymella* and an unclassified *Verrucariaceae* in the air samples (Fig. 4D). At the species level, more complete identification is needed.

### Fungal trophic modes and functional guilds

**Fig. 5:**
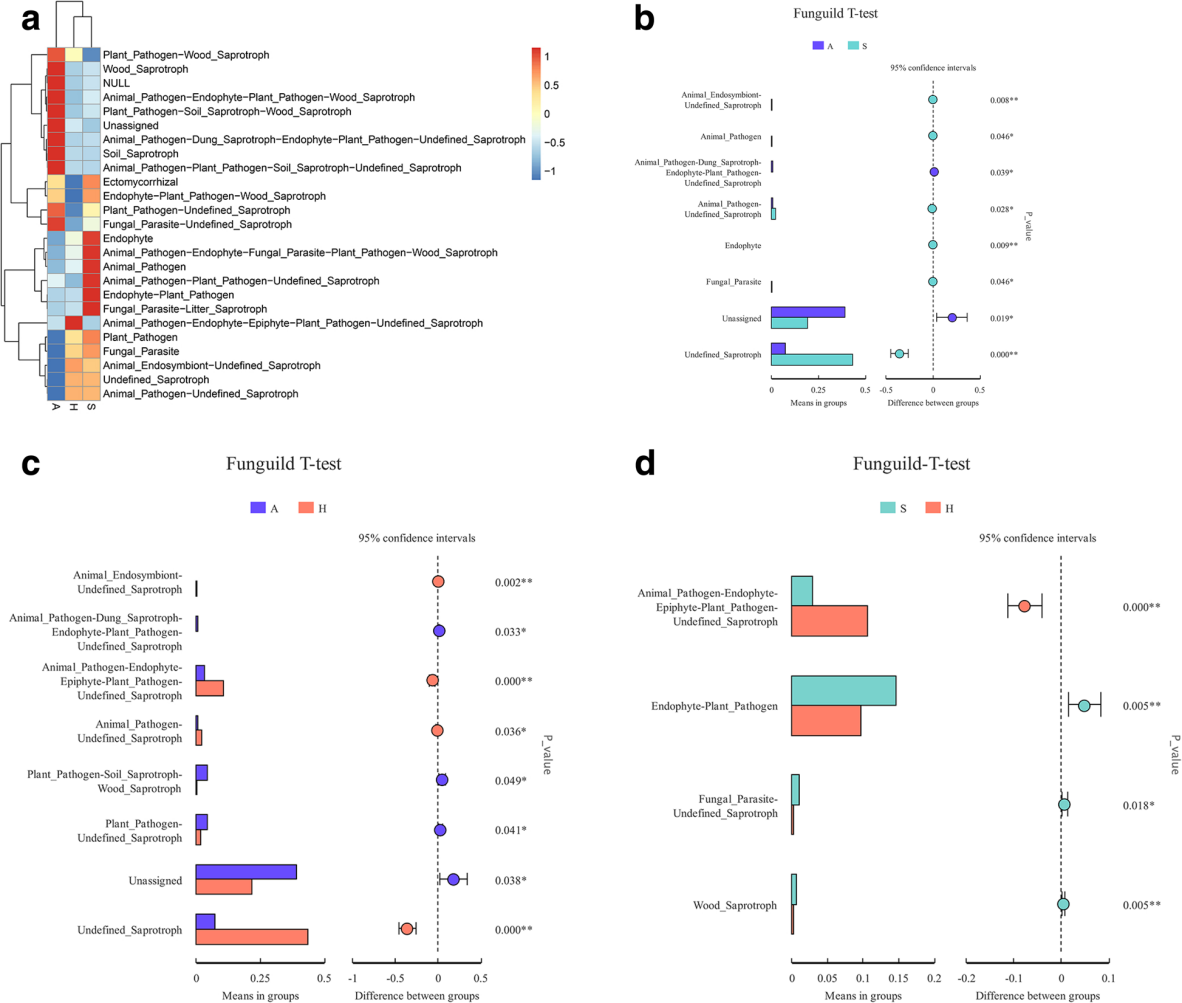
Fungal trophic modes and functional guilds in the air, handle, and saddle samples. Relative abundance of functional guilds (**A**). T-test of functional guilds (**B**-**D**). (A: air, H: handle, S: saddle)

FUNGuild was used to analyze the trophic modes and functional guilds of the fungal communities. Saprotrophs had an influential relative abundance of approximately 50% in bicycle surface specimens and approximately 1/8 in air specimens (see Additional Fig. 5). The heatmap representing functional taxonomy shows a high abundance in air, which is mostly nonoverlapping with that on shared bicycles (Fig. 5A). In the handle and saddle samples, the abundance of animal pathogens was obviously higher than that in the air samples (Fig. 5A). Compared with those in the saddle samples, more animal pathogens and fewer endophyte-plant pathogens were found in the handle samples (*P* < 0·05, Fig. 5B-D).

### Fungal co-occurrence networks

**Fig. 6:**
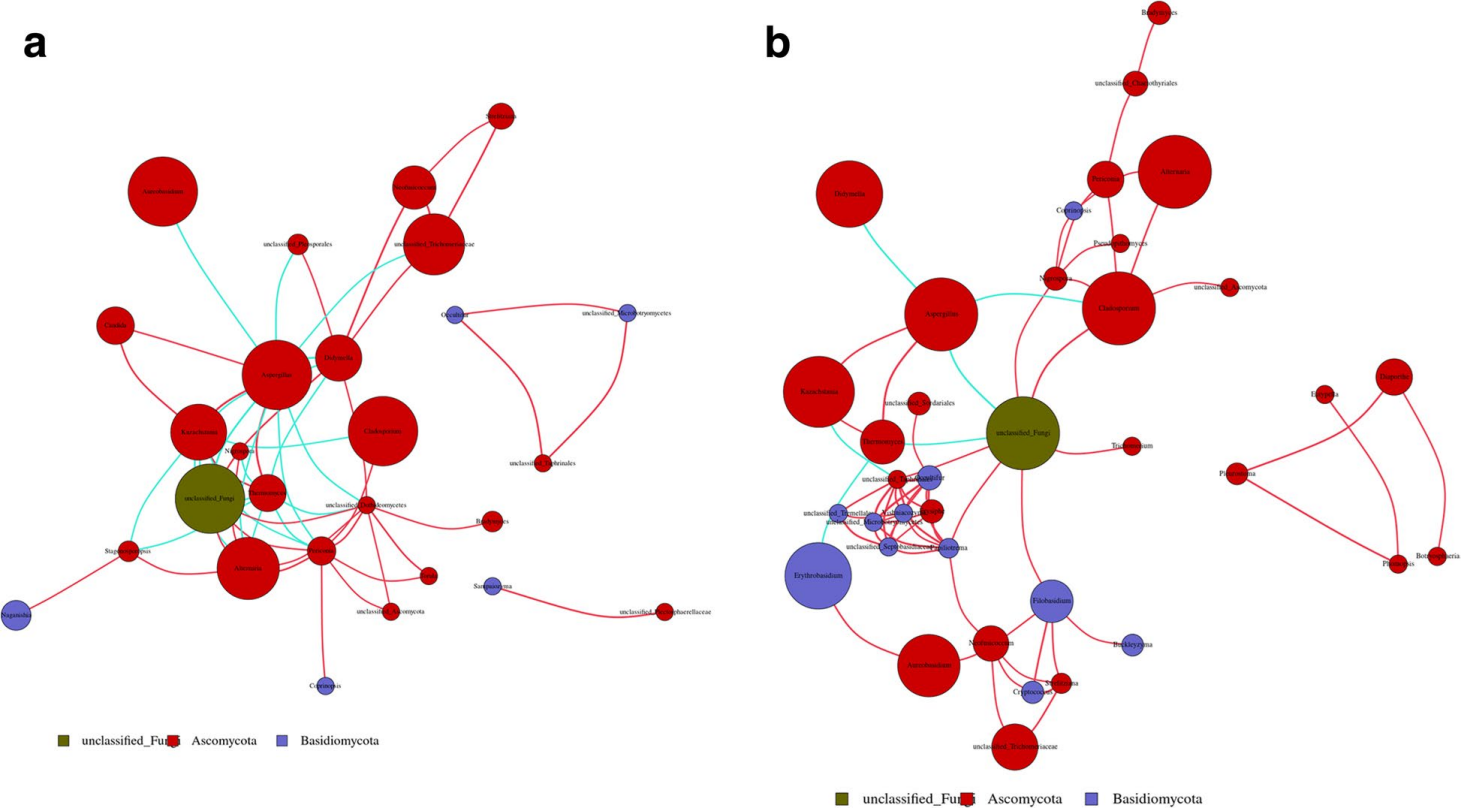
Fungal co-occurrence networks in the handle and saddle samples. Handle (**A**). Saddle (**B**). A strong and significant correlation (Spearman ρ < 0·6, *P* < 0·01) was detected. A node represents a fungal genus, and the size of the node is representative of the average relative abundance of the genus. The node colors represents phyla. Regarding the lines between two nodes, red represents a positive correlation, while blue represents a negative correlation

Correlation (ρ = 0·6) and *P* values (*P* = 0·01) were established. The fungal co-occurrence networks were obviously distinct between the shared-bicycle surfaces (Fig. 6A and B). Compared with the saddle specimens, the handle specimens had a larger clustering coefficient (CC: 50·5% vs. 48·8%) and a smaller average path length (APL: 2·18 vs. 3·03), indicating that the fungal communities on the handles were more closely connected than those on the saddles; the lower species richness was reconfirmed by the APL combined with a 0·2% smaller graph density.

Considering the statistical parameters, seven genera were enriched on handles. Two of the core genera, *Aspergillus* and *Kazachstania,* were mutually positively related, and both were associated with two subdominant genera (*Candida and Thermomyces*). *Aspergillus* was negatively correlated with four other enriched genera (unclassified fungi, *Alternaria*, *Aureobasidium*, and unclassified *Trichomeriaceae*) and two subdominant genera (*Periconia* and *Didymella*). *Kazachstania* had an inverse correlation with the dominant genera *Alternaria* and *Cladosporium* and unclassified fungi. However, lower relative abundances and greater mutual distances were present among genera of phylum Basidiomycota.

Eight genera were enriched on saddles. Among them, *Aspergillus* showed a negative relationship with *Didymella*, *Cladosporium*, and an unclassified fungi and a positive relationship with *Kazachstania* and the subdominant genus *Thermomyces*. *Cladosporium* was negatively correlated with *Alternaria* and an unclassified fungi. *Erythrobasidium,* as a highlighted genus of the phylum Basidiomycota, was positively correlated with *Aureobasidium* and negatively correlated with *Thermomyces.*

## Discussion

Our study identified differences in the compositions of fungal communities between the surfaces of shared bicycles and the surrounding air. The OTUs shared by the air deposition and bike surface specimens accounted for only 19·9% of the bike surface OTUs (Fig. 2A). Significant enrichment of the genera *Aspergillus*, *Kazachstania*, *Candida* and *Malassezia* was found on bicycles, probably because *Candida tropicalis* and *Malassezia globose* are resident fungi of human skin [7, 8]. *Aspergillus pseudoglaucus*, a common indoor fungus, has of osmo- and xero-characteristics and is widespread [9]. Skin and clothes contact may be the reason for their enrichment on shared-bicycle surfaces. *Kazachstania pintolopesii* has been isolated from forest soil and has a high minimum growth temperature of 20–43 °C [10]. Therefore, dust exposure under high-temperature conditions during summer may explain the high abundance of *Kazachstania pintolopesii*. Thus, we hypothesize that the major factor affecting the composition of the fungal community on the surface of shared bicycles is not the ambient air but the flora from the surface of the palm or clothing or road dust. Ambient temperature and humidity also have an effect on the microflora. However, the exact reasons for these results need to be further studied.

Regarding the fungal community structure, we found a significant difference between the saddle and handle samples. *Aureobasidium melanogenum* requires a relative humidity greater than 90% for proliferation, though it can tolerate extremely harsh conditions [11]. *Filobasidium magnum* requires a slightly acidic environment [12]. Their specific requirements for humidity and pH, respectively, may explain their higher abundance on handles, which are exposed to moist, sweaty hands, than on saddles. *Cladosporium tenuissimum* is an endophytic plant pathogen. It was more abundant on saddles than on handles, which was likely due to the shady trees in the parking lot.

According to the fungal co-occurrence networks, the fungal biota in the air samples were more complex and closely related than those in the shared-bicycle surface samples. This also indicated that fungi in the air had little influence on the fungal community of the bicycle surface. Among the bicycle samples, the fungal communities on the handles were more closely related, and those on the saddles were more intricate. These difference may be related to the ability of fungi to colonize different materials, the effects of human sweat and clothing, or the frequency at which the different parts of the shared bicycles were disinfected.

We assessed the pathogenicity of the most abundant species in our specimens (Table 1). *Aspergillus pseudoglaucus* had the highest absolute abundance in the handle and saddle samples, with overwhelming predominance, and the abundance was significantly higher than that in the air samples. It has been shown to be the most common indoor allergen [9], and sporadic cases of maxillary sinusitis and skin infection have been reported [13, 14]. *Aureobasidium melanogenum* has been reported to cause allergic pneumonia [27], cutaneous superficial infection at trauma sites and fungemia in children with lymphopenia [15, 16]. *Kazachstania pintolopesii* has been isolated from patients with leukoplakia [17]. *Filobasidium magnum* has been isolated from the nasal cavity of children with acute lymphoblastic leukemia and a male with a shoulder prosthesis [12, 18], and it sporadically causes vulvovaginitis and otomycosis [19, 28]. *Candida tropicalis* has been widely researched and found to reside on mucosal membranes rather than skin, with strong opportunistic pathogenicity and a high proportion of fluconazole-resistant properties [7, 20, 21]. The endometrium is also at risk of *Candida tropicalis* infection during pregnancy, and the skin is susceptible to hypoimmunity [22, 23]. Although *M. globose* is the most common fungus on human skin, it normally concentrates on the face and upper trunk due to its lipophilicity, and the hands play a core role in the process of fungal metastasis [8]. Their ectopic or excessive aggregation can lead to allergies or fungal infections [24–26], at the same time. FunGuild shows that animal pathogen has an obvious preponderance on the surface of shared bicycles, especially on the handles. Therefore, it is recommended that users (especially those with trauma, allergy, chronic disease, immunosuppression or immunodeficiency) first sterilize the handles and seat before using a shared bicycle. The timely cleaning and disinfection of the hands and clothes should not be neglected. It also suggests that governments should carry out regular and effective disinfection of shared bicycles to avoid the potential spread of diseases and allergens, especially during hot and humid summers.

**Table 1:** Literature review on the pathogenicity of different fungi among handle, saddle and air specimens

Comparison	Species	Guild	Year (reference)	Affected area/disease	Positive rate^a^	Age (year)/ gender	Risk factors
(H & Z) > A	*Aspergillus pseudoglaucus*	Animal Pathogen	1989 [13]	maxillary sinus	..	51/male	prolonged exposure to grain dust
			2012 [14]	skin(2), nail(3)/CAS	5/178	..	..
H > (Z & A)	*Aureobasidium melanogenum*	NA	2019 [14]	lung	..	79/male	contaminated bagpipe
			2016 [15]	face	..	46/male	NA
			2011 [16]	blood/fungemia	..	11/male	PLE, lymphopenia
(H & Z) > A	*Kazachstania pintolopesii*	NA	1987 [17]	oral/Leukoplakia	1/36	..	..
H > (Z & A)	*Filobasidium magnum*	NA	2011 [12]	nasal cavities	..	..	acute lymphoblastic leukemia
			2020 [18]	shoulder	..	68/male	Total Shoulder Arthroplasty
			2018 [18]	vaginal discharge	..	23/female	intra-uterine device
			2019 [19]	ear/otomycosis	..	35/female	ear canal trauma
(H & Z) > A	*Candida tropicalis*	Animal Pathogen-Endophyte-Undefined Saprotroph	2007 [20]	oral/Candidiasis	6/47	..	cancer
			2019 [21]	vagina/Candida vaginitis	35/197	..	..
			2011 [22]	chorion	..	28/female	pregnancy,cervical cerclage
			2020 [23]	skin/ Granuloma	..	57/male	lymphocytopenia
(H & Z) > A	*Malassezia globosa*	Animal Pathogen-Endophyte-Undefined Saprotroph	[24]..	skin	..	..	Excessive sebum secretion
			2014 [25]	oral	..	..	..
			2015 [26]	nasal vestibule	..	..	..

*H* Handle, *S* Saddle, *A* Air, *NA* No data available

^a^: The detection rate in the corresponding disease specimens. *CAS* Clinical Aspergillus strains was further identified by sequencing, *PLE* Protein Losing Enteropathy

We compared our fungal species of the handles with the top ten most abundant eukaryotes of palm from a review of human skin microorganisms [ 29]. *Malassezia globose, Candida parapsilosis* and *Malassezia sympodialis* coexisted in the handle group and human palms. *Malassezia globose* is the second most abundant fungus on the palm but ranked eighteenth in relative abundance on shared-bicycle handles. The other two coexistent species were detected in relatively small quantities in the handle samples. This suggests that the main source of these three fungi may be direct contact with human palms. However, such a large difference in the fungal community between palms and handles could be due to research bias or the interaction of more complex factors, such as handle temperature, humidity, hand disinfection and afforest environments.

Healthy people are generally immune to most pathogenic fungi. Moreover, *Trichophyton, Microsporum,* and *Epidermophyton*, which commonly cause cutaneous mycoses, were not discovered. This was probably due to (i) the small sample size; (ii) specimen collection during the rainy season, causing some of the fungi to be washed off from the bicycle by heavy rain, with the few remaining reads removed in the data quality control stage; and (iii) the need for strict environmental conditions (temperature or pH) or culture media with specific nutrition.

The limitations and future directions are as follows. (i) The UNITs database at the species level is still incomplete for fungal identification. (ii) ITS sequencing is based on genes and does not indicate fungal survival status. (iii) Specimens were collected in July, representing only the characteristics of shared-bicycle flora in summer. Additional studies with larger sample sizes are needed to further support our conclusion. The detection of viruses and bacteria in addition to fungi can be used to analyze microbial community structures in more dimensions. The correlations of environmental factors (season and weather, temperature, humidity, etc.) with microorganisms should be assessed.

## Conclusion

Our study analyzed fungal community structures in shared-bicycle surface (handles and saddles) samples; these communities were obviously different from those in the air deposition specimens, and more animal pathogens were present on bicycles than in air. In addition, we analyzed the possible factors leading to these differences and reviewed the pathogenicity of some significantly different species. Thus, governments around the world with shared-bicycle programs, bicycle program managers, and the public are reminded to pay attention to shared-bicycle hygiene.

## Supplementary Information

**Additional file 1: Figure 1**. Ternary plot of fungi among the three groups. (A: air, H: handle, S: saddle).

**Additional file 2: Figure 2**. OTU rarefaction curve for the three groups.

**Additional file 3: Figure 3**. Fungal alpha diversity of shared bicycles and nearby air. Chao 1 analysis (A), ACE analysis (B) (Chao 1 and ACE indices are positively correlated with the richness of the fungal community).

**Additional file 4: Figure 4**. Fungal diversity comparison between shared bicycles and nearby air by t-tests at the species level (A-C). The top 12 significantly different species as confirmed by the MetaStat analysis (D).

**Additional file 5: Figure 5**. Relative abundances of fungal trophic modes among the three groups.

## Data Availability

The datasets used and/or analysed during the current study are available from the corresponding author on reasonable request.
