# Supplementary material for: Characterization of fungal communities on shared bicycles in Southwest China
Source: BMC Microbiol. 2021 Oct 18;21:283. doi: 10.1186/s12866-021-02338-4 (PMC8523008; doi:10.1186/s12866-021-02338-4)

Characterization of fungal communities on shared bicycles in Southwest China

Additional file

Additional figure 1. Ternaryplot of fungi among the three groups (A: air, H: handle, S: saddle.)

Dot represents the top 30 most abundant genera, and the dot size represents the relative abundance of the genus in the three groups. A point close to an Angle indicates that it has a relatively high proportion in the corresponding group.

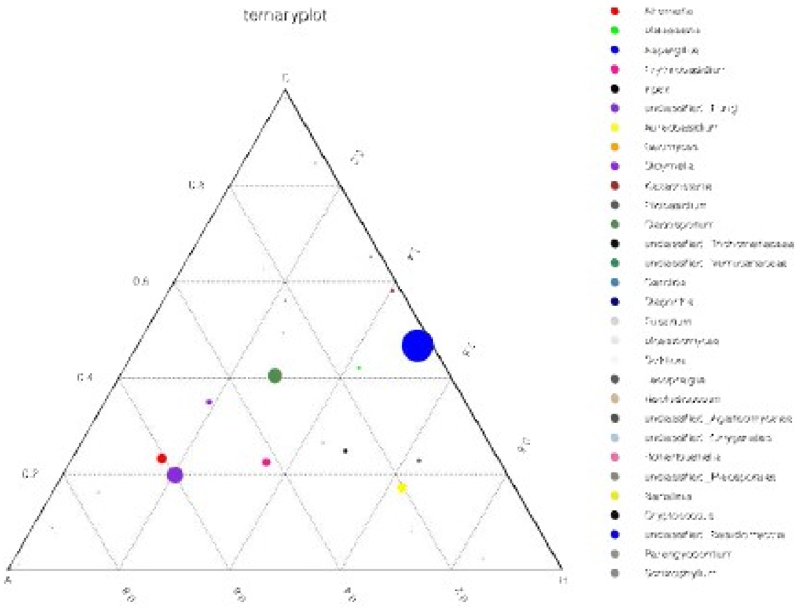

Supplement: Supplementary file 1 [file 12866_2021_2338_MOESM1_ESM.docx]
