# Supplementary material for: Characterization of fungal communities on shared bicycles in Southwest China
Source: BMC Microbiol. 2021 Oct 18;21:283. doi: 10.1186/s12866-021-02338-4 (PMC8523008; doi:10.1186/s12866-021-02338-4)

Additional figure 2. OTU rarefaction curve for the three groups. (A: air, H: handle, S: saddle.)

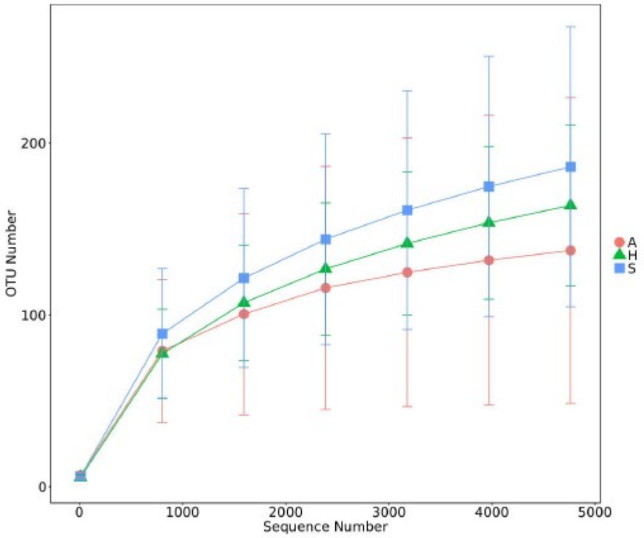

Supplement: Supplementary file 2 [file 12866_2021_2338_MOESM2_ESM.docx]
