# Supplementary material for: Characterization of fungal communities on shared bicycles in Southwest China
Source: BMC Microbiol. 2021 Oct 18;21:283. doi: 10.1186/s12866-021-02338-4 (PMC8523008; doi:10.1186/s12866-021-02338-4)

Additional figure 3. Fungal alpha diversity of shared bicycles and nearby air. Chao 1 analysis (A), ACE analysis (B) (Chao 1 and ACE indices are positively correlated with the richness of the fungal community). (A: air, H: handle, S: saddle.)

A:

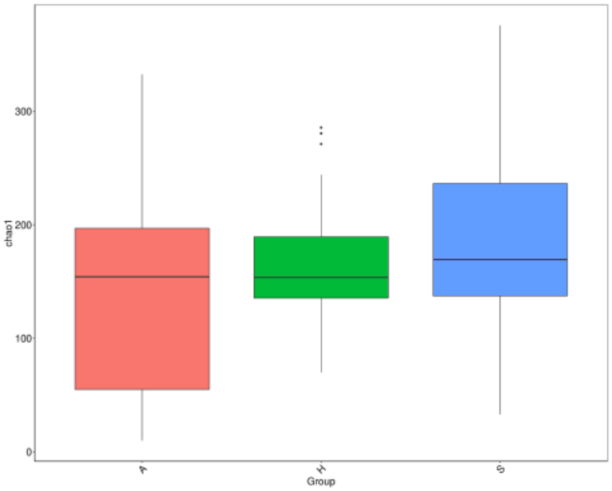

B:

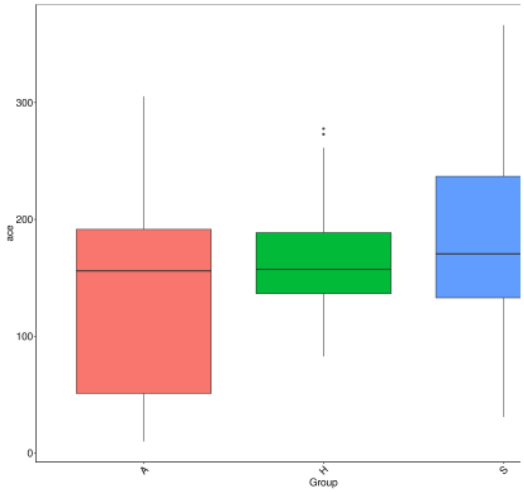

Supplement: Supplementary file 3 [file 12866_2021_2338_MOESM3_ESM.docx]
