# Supplementary material for: Characterization of fungal communities on shared bicycles in Southwest China
Source: BMC Microbiol. 2021 Oct 18;21:283. doi: 10.1186/s12866-021-02338-4 (PMC8523008; doi:10.1186/s12866-021-02338-4)

Additional figure 4. Fungal diversity comparison between shared bicycles and nearby air by t-tests at the species level (A-C). The top 12 significantly different species as confirmed by the MetaStat analysis (D). (A: air, H: handle, S: saddle)

A:

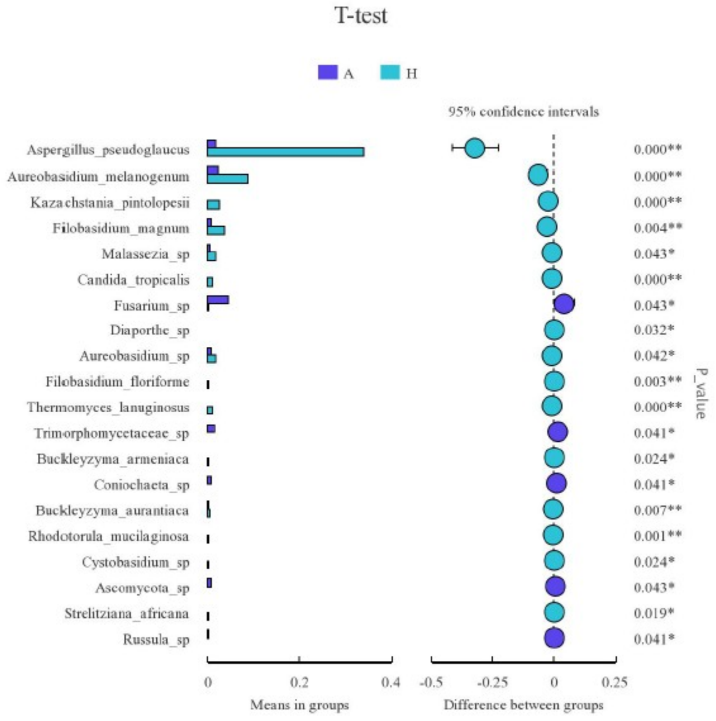

B:

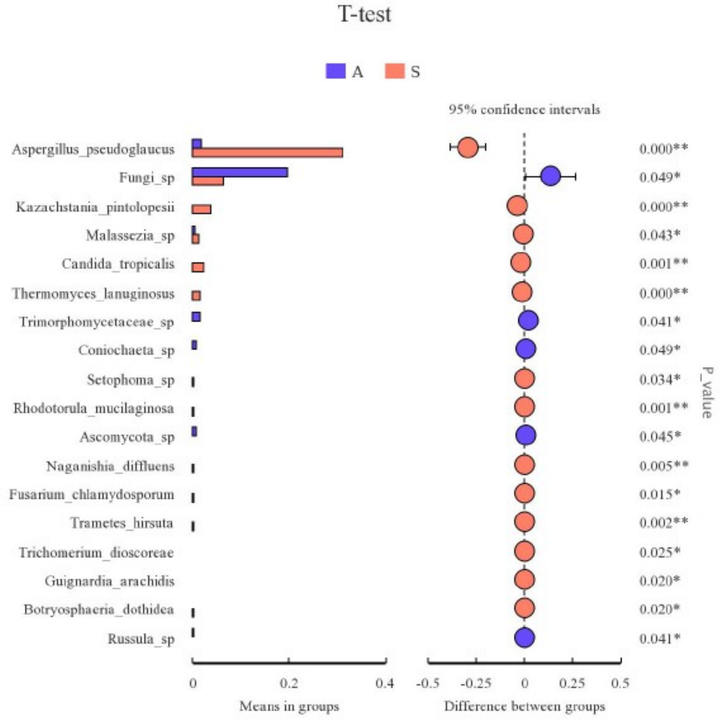
T-test
A
S
95% confidence intervals
Aspergillus_pseudoglaucus
Fungi_sp
Kazachstania_pintolopesii
Malassezia_sp
Candida_tropicalis
Thermomyces_lanuginosus
Trimorphomycetaceae_sp
Coniochaeta_sp
Setophoma_sp
Rhodotorula_mucilaginosa
Ascomycota_sp
Naganishia_diffluens
Fusarium_chlamydosporum
Trametes_hirsuta
Trichomerium_dioscoreae
Guignardia_arachidis
Botryosphaeria_dothidea
Russula_sp
0.000**
0.049*
0.000**
0.043*
0.001**
0.000**
0.041*
0.049*
0.034*
0.001**
0.045*
0.005**
0.015*
0.002**
0.025*
0.020*
0.020*
0.041*
P_value
0
0.2
0.4
Means in groups
-0.5
-0.25
0
0.25
0.5
Difference between groups

C:

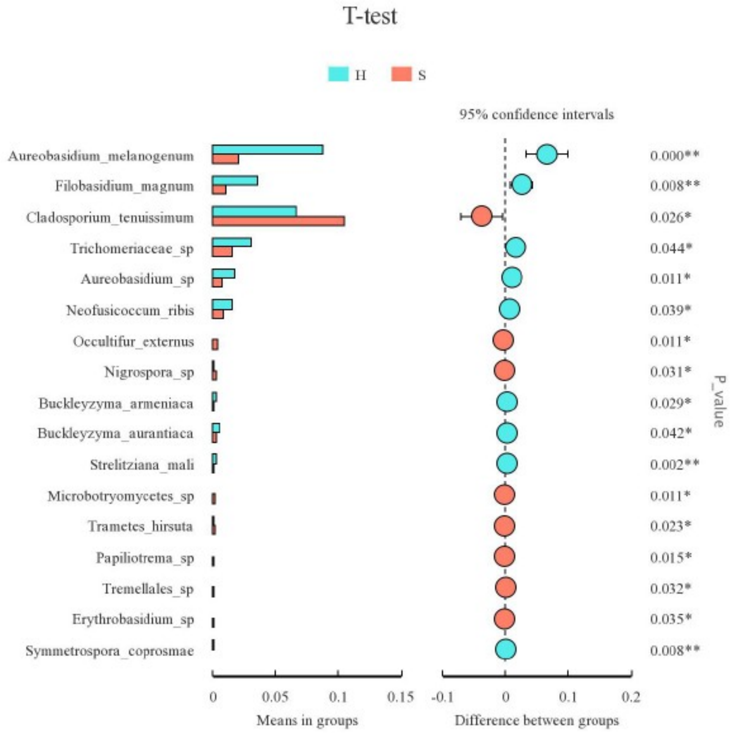

T-test
H
S
95% confidence intervals
Aureobasidium_melanogenum
Filobasidium_magnum
Cladosporium_tenuissimum
Trichomeriaceae_sp
Aureobasidium_sp
Neofusicoccum_ribis
Occultifur_externus
Nigrospora_sp
Buckleyzyma_armeniaca
Buckleyzyma_aurantiaca
Strelitziana_mali
Microbotryomycetes_sp
Trametes_hirsuta
Papiliotrema_sp
Tremellales_sp
Erythrobasidium_sp
Symmetrospora_coprosmae
0.000**
0.008**
0.026*
0.044*
0.011*
0.039*
0.011*
0.031*
0.029*
0.042*
0.002**
0.011*
0.023*
0.015*
0.032*
0.035*
0.008**
P_value
0
0.05
0.1
0.15
Means in groups
-0.1
0
0.1
0.2
Difference between groups

Means in groups Difference between groups

D:

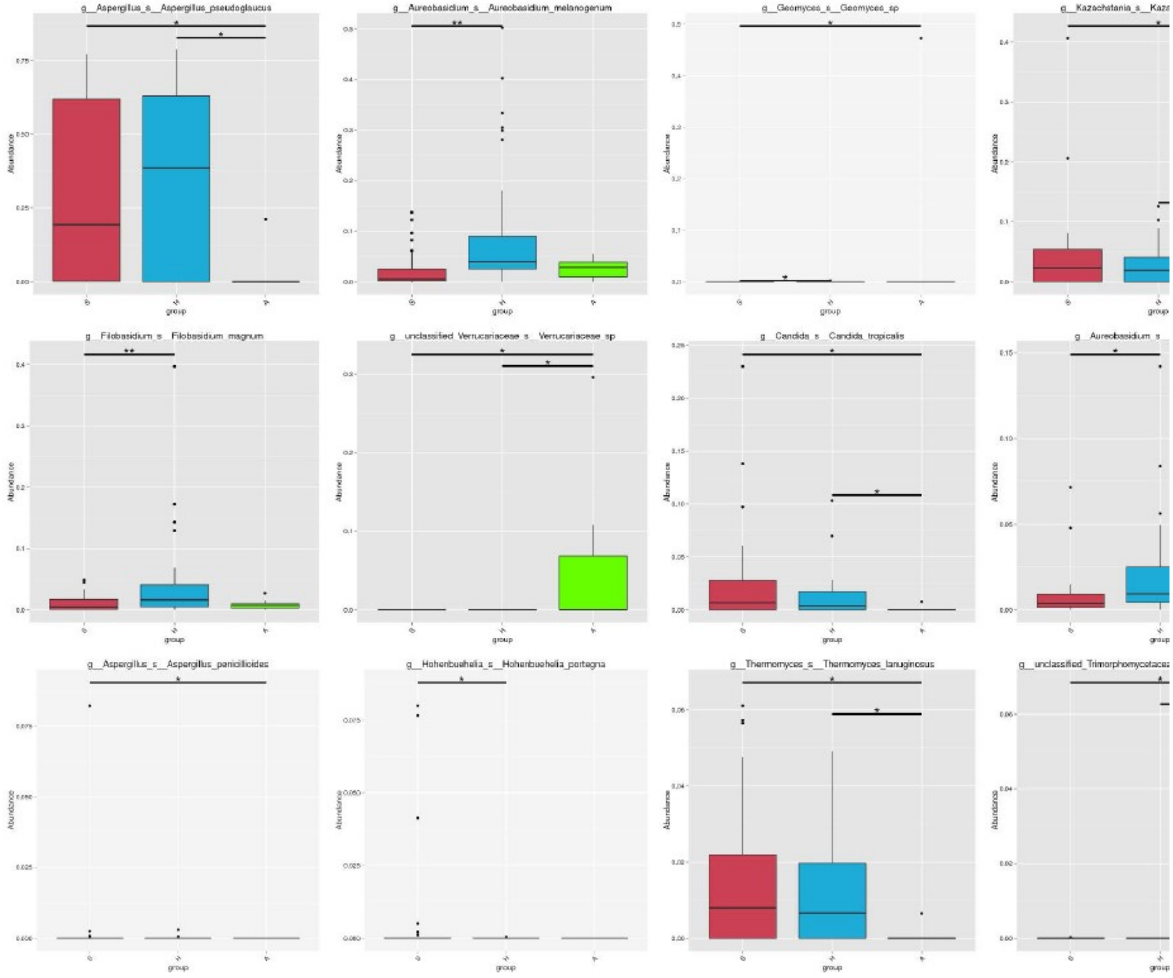

Supplement: Supplementary file 4 [file 12866_2021_2338_MOESM4_ESM.docx]
