# Supplementary material for: Characterization of fungal communities on shared bicycles in Southwest China
Source: BMC Microbiol. 2021 Oct 18;21:283. doi: 10.1186/s12866-021-02338-4 (PMC8523008; doi:10.1186/s12866-021-02338-4)

Additional figure 5. Relative abundances of fungal trophic modes among the three groups. (A: air, H: hand

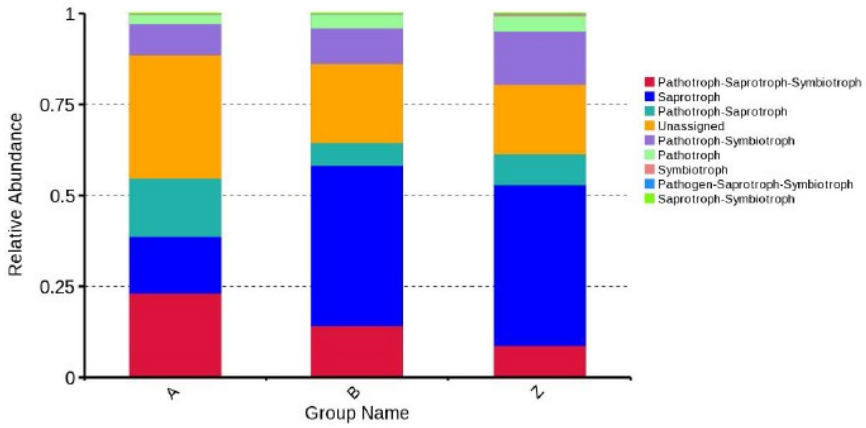

Supplement: Supplementary file 5 [file 12866_2021_2338_MOESM5_ESM.docx]
